# When Bacterial Culture Fails, Metagenomics Can Help: A Case of Chronic Hepatic Brucelloma Assessed by Next-Generation Sequencing

**DOI:** 10.3389/fmicb.2018.01566

**Published:** 2018-07-17

**Authors:** Vladimir Lazarevic, Nadia Gaïa, Myriam Girard, Stefano Leo, Abdessalam Cherkaoui, Gesuele Renzi, Stéphane Emonet, Sharon Jamme, Etienne Ruppé, Sandrine Vijgen, Laura Rubbia-Brandt, Christian Toso, Jacques Schrenzel

**Affiliations:** ^1^Genomic Research Laboratory, Division of Infectious Diseases, Faculty of Medicine, Geneva University Hospitals, Geneva, Switzerland; ^2^Bacteriology Laboratory, Department of Genetics and Laboratory Medicine, Faculty of Medicine, Geneva University Hospitals, Geneva, Switzerland; ^3^Department of Community, Primary Care and Emergency Medicine, Faculty of Medicine, Geneva University Hospitals, Geneva, Switzerland; ^4^Division of Clinical Pathology, Department of Genetics and Laboratory Medicine, Faculty of Medicine, Geneva University Hospitals, Geneva, Switzerland; ^5^Department of Surgery, Faculty of Medicine, Geneva University Hospitals, Geneva, Switzerland

**Keywords:** *Brucella*, liver abscess, clinical metagenomics, molecular diagnosis of infectious diseases, next-generation sequencing

## Abstract

Here, we sequenced DNA extracted from a necrotic hepatic lesion from a patient with suspected chronic hepatic brucelloma but negative culture results. Although most of the taxonomically classified sequencing reads corresponded to human genome sequences, our data suggest that whole-metagenome shotgun sequencing may be used together with other tests to strengthen the diagnosis of hepatic brucelloma.

## Background

The genus *Brucella*, characterized by Gram-negative, non-spore-forming, slow-growing bacteria, contains 12 described species and many isolates of human and animal origin that have not yet been formally taxonomically described (Whatmore et al., 2016). Human infections caused by *Brucella* (called brucellosis) are among the most common zoonoses worldwide with an estimated 500,000 cases each year (Pappas et al., 2006).

After entering the human body, *Brucella* is phagocytized, intracellularly replicated and hematogenously disseminated to all lymphoreticular organs, including the liver (von Bargen et al., 2012). Hepatic abscess, as a complication of chronic brucellosis, is rarely encountered in clinical practice. Barutta et al. (2013) reviewed 60 cases of hepatic brucelloma in the medical literature spanning the years 1904–2013 and found that most cases (57%) were reported in the Mediterranean countries.

Isolation of *Brucella* species requires strict biosafety measures (level 3), as these species are the predominant cause of laboratory-acquired infections (Singh, 2009). Brucellosis often remains undiagnosed by culture-based analysis of clinical specimens such as blood (Yagupsky, 1999), hepatic abscess (Agostinelli et al., 2012), or infected heart valves (Moter et al., 2002), but may be detected by serological tests, which, in some cases, can also be difficult to interpret (Díaz et al., 2006). Molecular methods based on the polymerase chain reaction (PCR), including quantitative PCR (qPCR) assays, have been developed to increase the sensitivity and specificity of the detection of *Brucella* in bacterial cultures, sera, blood, and other tissues (Yu and Nielsen, 2010).

Clinical metagenomics relies on the use of next-generation sequencing and bioinformatics tools for identifying DNA fragments from pathogenic microorganisms (Rose et al., 2015; Ruppé et al., 2016; Forbes et al., 2017). Despite some challenges such as incomplete genomic sequence databases and difficulties in determining limits of detection, clinical metagenomics analyses have proven promising for a variety of clinical samples, most notably in the detection of difficult-to-culture infectious agents. It is anticipated that clinical metagenomics analyses will be widely implemented in routine diagnostic testing within the next 10 years (Forbes et al., 2017).

Here, we performed whole-metagenome shotgun sequencing (WMGS) of DNA extracted from a hepatic surgical specimen collected from a patient with suspected chronic hepatic brucelloma to test this emerging diagnostic approach that does not focus on specific bacterial species.

## Materials and Methods

### Ethics Statement

According to hospital protocol, no formal ethics approval was required for this study. The patient agreed and provided written informed consent for publication of this Case Report and any accompanying images.

### Culture Methods

Abscess drainage fluid and surgically obtained liver specimens were sent to the bacteriology laboratory for Gram-staining and immediate inoculation onto Columbia blood agar (incubated in a CO_2_-enriched atmosphere), chocolate agar (incubated in a CO_2_-enriched atmosphere), CDC anaerobe 5% sheep blood agar (incubated under anaerobic conditions), and brain–heart infusion broth. The solid media and brain–heart infusion broth were incubated for 1 and 3 weeks, respectively, at 37°C. Blood cultures were processed by using the BD BACTEC^TM^ FX Blood Culture System (Becton Dickinson).

### *Brucella*-Specific qPCR Assay

Abscess drainage fluid was analyzed using a qPCR-based assay targeting insertion sequence IS*711*, which is unique to the genus *Brucella* (Hinić et al., 2008). DNA was extracted using a MagNA Pure LC instrument and a MagNA Pure LC DNA Isolation Kit II (Roche Molecular Biochemicals) according to the manufacturer’s instructions. TaqMan Universal PCR Master Mix with AmpErase UNG (Applied Biosystems) was used along with 0.5 μL of input DNA and nuclease-free water (Promega). Each PCR assay was performed in triplicate.

### Serological Tests for Brucellosis

Serum samples from the patient were sent to CERBA Laboratory (Cergy-Pontoise) for serological analysis using the Rose Bengal (Morgan et al., 1969) and Wright serum agglutination tests (Wright and Semple, 1897), which, essentially detect IgG and IgM antibodies (Sanofi Diagnostics Pasteur reagents), respectively. In addition, indirect immunofluorescence (IIF) analysis (IIF Laboratoire CERBA reagents), was performed for IgG and IgM.

### Histopathological Examination of the Necrotized Liver Tissue

After a careful gross examination of the resected hepatic specimen, a large sampling of the necrotic hepatic lesion was performed. A portion of the sample was frozen for later use, while the other portion was used for histological evaluation. To prepare the sample for histological examination, the tissue was fixed in 4% formaldehyde, dehydrated using increasing concentrations of alcohol, and then cleared with xylene. The tissue was then embedded in paraffin and cut into 4-μm thick sections that were stained with haematoxylin and eosin. Gram and periodic acid Schiff (PAS) staining was also performed on some samples.

### DNA Extraction

A portion of the frozen necrotized hepatic tissue was cut into small pieces using a scalpel. DNA was extracted from 65 mg of shredded sample using an Ultra-Deep Microbiome Prep Kit (Molzym, Bremen, Germany) according to the manufacturer’s instructions (Version 2.0) for tissue samples. This method aims to decrease human DNA content via differential lysis of human and bacterial/fungal cells, followed by the elimination of free DNA including that released from human cells. For DNA extraction without selective enrichment of bacterial/fungal DNA, another 30-mg aliquot of shredded sample was mixed with 550 μL of GT Buffer (RBC Bioscience). The mixture was shaken in a NucleoSpin Bead Tube (Macherey-Nagel) containing ceramic beads for 20 min at maximum speed on a Vortex-Genie 2 with a horizontal tube holder (Scientific Industries). A 1-μl aliquot of a 50 mg/mL solution of RNAse A (from bovine pancreas, Sigma-Aldrich ref 10109142001) was added to the sample, which was then incubated at room temperature for 5 min. The lysate was centrifuged at 11,000 × *g* for 2 min and a 400-μL aliquot of the supernatant was loaded into the sampling tube of a MagCore Sample Tube (RBC Bioscience) of the MagCore HF16 Automated Nucleic Acid Extractor (RBC Bioscience). DNA was purified using a MagCore Genomic DNA Tissue Kit (RBC Bioscience) with program 401, eluted in 60 μL of Tris–HCl (10 mM, pH = 8) and stored at -20°C.

Negative extraction controls (without addition of a clinical sample) were performed for both DNA extraction protocols.

### qPCR Assays

The concentrations of bacterial and human DNA were determined by qPCR experiments as described previously (Lazarevic et al., 2014) using 16S rRNA and β-actin reference genes, respectively. Reference curves for bacterial and human DNA quantitation were generated using known concentrations of *Escherichia coli* DH5α genomic DNA and human genomic DNA from the TaqMan β-Actin Detection Reagent Kit (Applied Biosystems), respectively. For this assay, 1 ng of bacterial DNA determined using the *E*. *coli* DH5α standard, corresponded to 1493 copies of the 16S rRNA gene.

### Broad-Range 16S rRNA PCR for Identification of Bacteria

One ng of purified DNA (in a total volume of 5 μL) was sent to an ISO17025-certified diagnostic laboratory for bacterial identification by 16S rRNA amplification/sequencing.

### DNA Sequencing

Metagenomic libraries were prepared from 5 μL of purified DNA diluted at 0.2 ng/μL for clinical samples or from 5 μL of undiluted DNA extract for negative extraction controls using a Nextera XT DNA Sample Preparation Kit (Illumina). Samples were prepared as per the manufacturer’s instructions, except that 16 PCR enrichment cycles were used instead of 12. The libraries were sequenced for 2 × 250+8 cycles on an Illumina MiSeq instrument at Fasteris (Plan-les-Ouates, Switzerland) using a MiSeq Reagent Kit v.3 and MiSeq Control Software 2.6.2.1 (Illumina). Demultiplexed fastq files were generated with CASAVA-1.8.2 from on-instrument base-calling using Real-Time Analysis (RTA) software v.1.18.54.0. The Trimmomatic package (Bolger et al., 2014) was used to remove bases that corresponded to the standard Illumina adapters and to trim low-quality ends of reads at the beginning of a 4-base-wide sliding window with an average Phred quality <5.

### Bioinformatics Analysis

Reads with a low-quality base score of Q10 or a mean quality score of Q30 over a sliding 20-base window were further trimmed using the Mothur v1.35.1 trim.seqs command (Schloss et al., 2009). Any read that, after trimming, had a length <150 bases was discarded together with its corresponding paired read. To filter out putative artificial replicate reads, we used a home-made script (available upon request) that retains only the longest sequence reads showing 100% identity over the first 100 bases in either the forward or reverse direction. Remaining reads were classified using Kraken (Davis et al., 2013) v.0.10.5-beta using default parameters. After filtering out the read pairs that matched the human genome, sequencing data were deposited in the European Nucleotide Archive^1^ under project number ERP022764 (PRJEB20595). Reads classified by Kraken as belonging to genus *Brucella* were mapped to the two chromosomes of *Brucella melitensis* 16M (Minogue et al., 2014) using BWA (Li and Durbin, 2009) v.0.7.15-r1140 with the default parameters.

## Case Report

**FIGURE 1:**
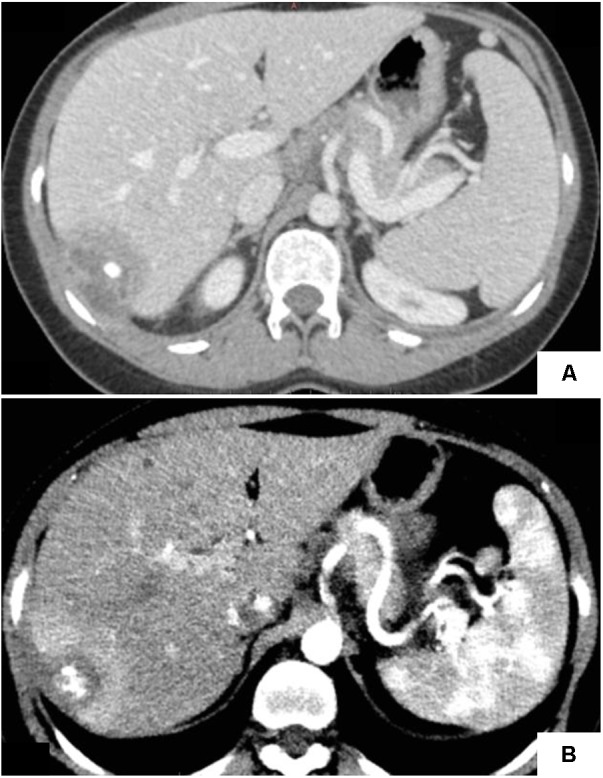
Abdominal CT-scans showing liver abscess. **(A)** Central calcification of the liver abscess observed in 2010, suggesting of a granulomatous infection. **(B)** The abscess observed in 2016 showing heterogeneous density and reduced size as compared to 2010.

Here we report the case of a Portuguese woman, first admitted in 2010 at the age of 29 years, with a 1-month history pain in the right shoulder and the right superior quadrant of the abdomen 4 months after uncomplicated delivery. Abdominal echography and CT scan (**Figure 1A**) revealed the presence of a 6 × 4-cm hepatic abscess with a central calcification suggestive of a brucelloma. The serology for *Brucella* spp. was positive, presenting IIF IgG titers of 1/320 and 1/160 at an 18-day interval. These antibodies are most often present in chronic brucellosis. Rose Bengal and Wright tests, performed only after a second sampling, were positive and negative, respectively. Echocardiography ruled out concomitant endocarditis. To avoid relapse, the abscess was surgically opened to remove the central calcification. Blood and abscess drainage fluid cultures were negative, whereas a (genus) *Brucella*-specific qPCR assay targeting IS*711* assay performed using abscess drainage fluid was positive. The patient was placed on a regimen of doxycycline and rifampicin for 3 months.

Six years later, the patient returned to our hospital with similar complaints of fever and pain in the right abdomen and shoulder. Abdominal echography and CT scan (**Figure 1B**) again showed an abscess in the same location with multiple calcifications inside. *Brucella*-specific qPCR assay on the abscess drainage fluid was positive (*C*t value = 31), while blood and abscess drainage fluid cultures were negative. Rose Bengal and Wright serological tests performed 5 days before partial hepatectomy, and again after a 54-day interval, were also negative. Our surgeon decided to perform an atypical hepatectomy of segments 6 and 7 to eradicate what he considered to be a relapse of a hepatic brucelloma. The surgically obtained purulent liver material was culture-negative. The patient received a combination of ceftriaxone and doxycycline but, because of an allergic reaction to ceftriaxone, the treatment was changed to gentamicin, doxycycline and rifampicin for 2 weeks, followed by another 3 months of doxycycline and rifampicin.

**FIGURE 2:**
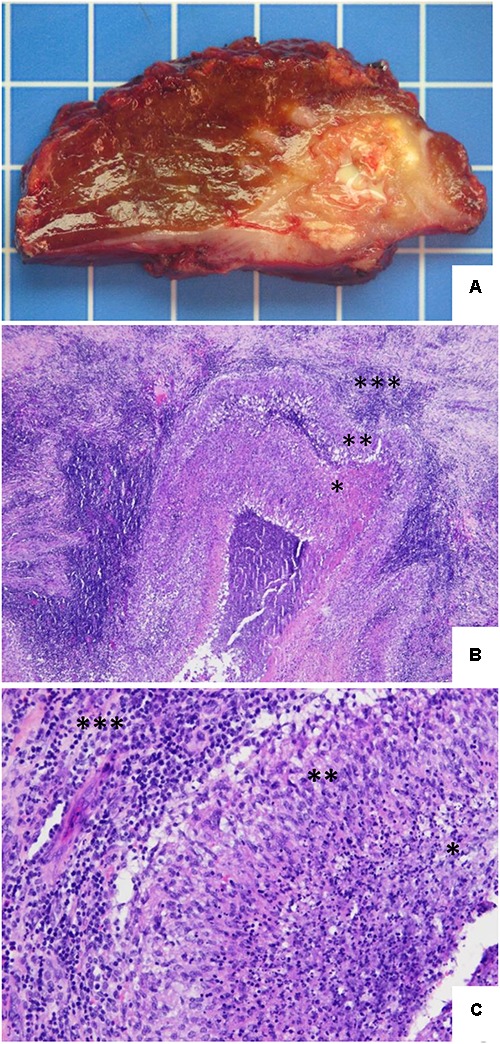
Hepatic histopathology of human brucellosis. **(A)** Gross section of the hepatic segments showing a large and relatively well-demarcated lesion with some calcifications and a central necrotic and suppurative area. One square = 1 cm^2^. **(B)** Hematoxylin and eosin stain. The lesion consists of a large pseudotumoral abscessed lesion with a central necrotic area (^∗^) surrounded by a serpiginous granulomatous inflammation (^∗∗^) and associated with abundant peripheral fibro-inflammatory tissue (^∗∗∗^) (original magnification, ×4). **(C)** Hematoxylin and eosin stain. Higher power view illustrating the transition between the central suppurative necrosis (^∗^), delineated by a rim of epithelioid histiocytes arranged in palisade (^∗∗^), with peripheral fibrous tissue comprising an intense lymphoplasmocytic infiltrate (^∗∗∗^) (original magnification, ×20).

Gross evaluation of the resected hepatic specimen taken in 2016 showed a unique and partially well-demarcated lesion measuring 3 cm × 2.5 cm × 2 cm, containing a central necrotic area (**Figure 2A**). Microscopic examination revealed that this lesion consisted of a large epithelioid cell granuloma with some calcifications and abundant suppurative necrosis (**Figures 2B,C**). No pathogens were identified by PAS and Gram staining. The lesion appeared to occur through the fusion of granulomas, resulting in the development of suppurative necrosis and leading to a true abscess caused by the persistent presence of brucellae within macrophages. Histologically, similar findings have been observed in cases of tuberculosis, yersiniosis, and tularaemia.

We extracted DNA from the hepatic surgical specimen upon relapse in 2016 using two methods: (i) without microbial DNA enrichment, recovering both free and cellular DNA released by mechanical lysis and (ii) with bacterial/fungal DNA enrichment by including degradation of extracellular DNA and DNA released from selectively lysed host cells. The routine broad-range PCR test for detection and identification of bacterial pathogens in clinical specimens was negative for DNA extracts obtained by either extraction procedure. Human and bacterial DNA loads in DNA extracts were determined by qPCR analyses. The concentration of human DNA was 4.7 and 104.2 ng/μL in enriched and unenriched extracts, respectively, which was roughly 1.1 × 10^5^ and 1.8 × 10^6^ times higher than the estimated concentration of bacterial DNA.

**FIGURE 3:**
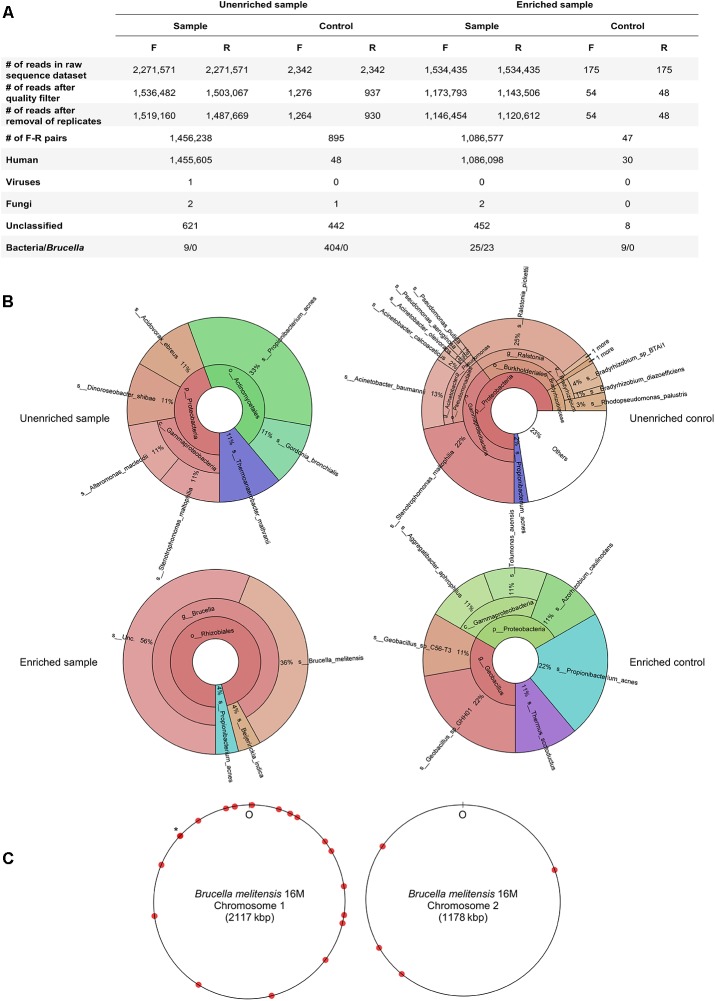
Number and taxonomic assignments of sequence reads obtained by WMGS of DNA extracts from hepatic biopsy samples and negative controls. **(A)** Number of sequence reads at different steps of data analysis. Taxonomic assignments were made using Kraken. F, forward reads; R, reverse reads. **(B)** Krona plots (Ondov et al., 2011) of bacterial taxa identified by Kraken. Taxa comprising <1% of the unenriched sample were pooled and represented as “others.” **(C)** Mapping of reads on the *B. melitensis* 16M chromosomes. The chromosome regions to which pairs of sequence reads were mapped are indicated by red circles. Two non-overlapping DNA fragments that map close to each other are depicted by an asterisk.

Analysis of WMGS data confirmed the abundance of human DNA in the clinical sample and showed a 3.7-fold increase in the proportion of reads assigned to bacteria in the enriched sample compared with the unenriched sample (**Figure 3A**). Of 25 (paired) reads classified by Kraken as bacterial in the dataset of the enriched sample (**Figure 3A**), 23 were assigned to the genus *Brucella*, while two paired reads corresponded to known reagent contaminants *Beijerinckia* and *Propionibacterium* (Salter et al., 2014; **Figure 3B**). We retrieved 23 paired reads corresponding to the genus *Brucella* and mapped them to the *B*. *melitensis* 16M genome. Nineteen of the paired reads mapped to the chromosome 1, while four mapped to chromosome 2, the smaller of the two chromosomes (**Figure 3C**). Both the forward and reverse reads of the read pairs showed 100% nucleotide sequence identity to the *B*. *melitensis* 16M genome, except for one read, showing six single nucleotide mismatches (96% identity). Thirteen individual reads (forward or reverse) showed 100% identity to *B. melitensis* strains only, while 32 reads showed 100% identity to *Brucella* species, including *B*. *melitensis* (data not shown). The length of the metagenomic fragments that mapped to the reference *B*. *melitensis* 16M genome was estimated to be 168–537 bp. All but one of these fragments were located either within (13) or partly overlapping with (9) the open reading frames. The mapped fragments showed no obvious distribution pattern except that they were slightly over-represented in the half of the chromosome 1 containing the origin of replication.

In contrast to the enriched sample, from which most (23/25) of the bacterial paired reads corresponded to *Brucella*, none of the nine bacterial paired reads identified in the unenriched sample dataset mapped to the genus *Brucella* (**Figure 3B**). This difference may be ascribed to a lower amount of biopsy material processed with the combined bead beating and MagCore DNA extraction protocol, or to a higher degree of exogenous DNA contamination using this extraction procedure. In the unenriched sample dataset, *Propionibacterium*, a common reagent contaminant, was the most abundant bacterial taxon; however, it was only represented by three sequences. Other bacteria identified by WMGS in the unenriched sample DNA extract also corresponded to known (*Acidovorax* and *Stenotrophomonas*) or likely (marine bacterium *Alteromonas macleodii*, algal symbiont *Dinoroseobacter* and *Thermoanaerobacter*) reagent contaminants. Only a *Gordonia* sequence corresponded to a putative human pathogen.

Sequencing of DNA extracts from the negative controls produced few reads (**Figure 3A**). The only bacterial genus identified in both the enriched and unenriched negative controls were *Propionibacterium*, which was also the only genus found in both DNA extracts of the hepatic specimen. Analysis of the dataset obtained from the unenriched negative extraction control revealed other taxa typically associated with reagent contamination, including *Stenotrophomonas, Acinetobacter, Ralstonia*, and *Bradyrhizobium*. Bacterial reads from the enriched negative extraction control mapped to several environment-associated taxa, including *Geobacillus, Tolumonas, Azorhizobium*, and *Thermus*, as well as to *Aggregatibacter aphrophilus*, an indigenous member of the human oropharyngeal microbiota.

## Discussion

This case is remarkable for three reasons: the typical picture obtained from the imaging analysis, the clinical relapse after a 6-year interval, and negative Rose Bengal and Wright serologies at relapse. Our hypothesis of relapsed brucelloma relied on: (i) the patient’s complaints of fever and pain in the right abdomen and shoulder; (ii) the observation of hepatic pathological changes that occurred at the site of surgically removed primary lesions; and (iii) a positive *Brucella*-specific qPCR result. In addition, the analysis of the WMGS data, the first obtained from a hepatic brucelloma sample, suggested the presence of *B*. *melitensis*. It seems likely that the organism persisted in the envelope of the abscess and slowly multiplied *in situ* between 2010 and 2016, leading to the clinical relapse.

Because members of the genus *Brucella* are not recognized as common reagent contaminants (Salter et al., 2014), and as *Brucella* was the dominant genus in the enriched sample but was absent in the negative controls, the possibility that it originated from an exogenous source seems unlikely. In addition, the bacterial DNA enrichment procedure that we used included a DNase treatment prior to bacterial lysis, which removes extracellular DNA and DNA from dead cells present in the sample (Pezzulo et al., 2013). We cannot formally exclude the possibility that brucellae in the hepatic sample taken from the patient were dead. However, it seems more likely that the bacteria were alive, at least in the days preceding the sampling date, as experiments conducted in an animal model showed that DNA from dead bacteria is cleared from the host within days (Straubinger, 2000).

While the presence of sequence reads assigned to pathogens have a high positive predictive value, the exclusion of an infectious process, based on the absence of such sequences, is clearly not sufficient. However, WMGS analysis of multiple biopsy specimens of the same lesion may increase the likelihood of pathogen identification. In this study, we used less than 10% of the capacity of the MiSeq platform (currently about 25 million paired reads) for each enriched and unenriched sample. A higher sequencing depth may be achieved using the full capacity of current sequencing platforms, and more critically, better removal of human DNA would allow us to obtain draft genomic sequences of pathogens from different types of clinical samples. From that perspective, WMGS may be used not only for identification of bacteria, but also for bacterial typing, circumventing the need for bacterial culture and use of multiple specific PCR or qPCR assays.

## Concluding Remarks

To the best of our knowledge, this is the first report of the use of WMGS on a hepatic brucelloma sample. Our data suggest that WMGS can provide an alternative or supplement to bacterial cultures and other currently available molecular tests for diagnosing hepatic brucelloma and other abscesses. Early detection of asymptomatic relapses of brucellosis is challenging and requires post-treatment follow-up, including peripheral blood culture and PCR, serological tests and imaging (Morata et al., 1999; Díaz et al., 2006).

## Author Contributions

SE, SJ, SV, LR-B, CT, and JS analyzed and interpreted the patient data. MG, AC, and GR performed the experiments. NG, SL, ER, and VL analyzed the metagenomics data. VL, SE, SV, LR-B, CT, and JS wrote the manuscript. All authors read and approved the final manuscript.

## Conflict of Interest Statement

The authors declare that the research was conducted in the absence of any commercial or financial relationships that could be construed as a potential conflict of interest.

## Footnotes

1 www.ebi.ac.uk/ena
